# Supplementary material for: The Regulatory Effects of Citrus Peel Powder on Liver Metabolites and Gut Flora in Mice with Non-Alcoholic Fatty Liver Disease (NAFLD)
Source: Foods. 2021 Dec 6;10(12):3022. doi: 10.3390/foods10123022 (PMC8701357; doi:10.3390/foods10123022)
Supplement: Supplementary file 1 [file foods-10-03022-s001.zip › foods-1428786-supplementary.pdf]

Table S1. Bioactive ingredients in Jinggang pomelo and Gannan navel orange peel (ND: undetected).

Compounds	*Jinggang* pomelo peel	*Gannan* navel orange peel
Pectin (%)	18.5	19.3
Essential oil (mL/100g)	0.16	0.20
Limonene (%, essential oil)	45.5	41.7
Polyphenol (μg/g)	3825.55	2912.37
Narirutin (μg/g)	2359.44	1105.37
Naringin (μg/g)	851.43	480.37
Neohesperidin (μg/g)	2.77	257.95
Hesperidin (μg/g)	1.94	239.62
Diosmin (μg/g)	88.86	110.99
Isoferulic acid (μg/g)	10.08	102.21
Ferulic acid (μg/g)	8.06	101.08
Eriocitri (μg/g)	16.74	65.23
Vanillic acid (μg/g)	17.19	17.78
P-coumaric acid (μg/g)	0.93	11.32
Gallic acid (μg/g)	ND	6.93
Sinensetin (μg/g)	16.49	6.43
4-hydroxybenzoic acid (μg/g)	5.70	5.52
Hesperetin (μg/g)	ND	2.50
Hippuric acid (μg/g)	0.88	0.88
4-hydroxyphenylacetic acid (μg/g)	16.59	ND
Phloretic acid (μg/g)	3.57	ND
Naringenin (μg/g)	0.96	ND
